# Supplementary material for: What does it take to learn the rules of RNA base pairing? A lot less than you may think
Source: bioRxiv. 2025 Aug 2:2025.07.31.668042. Preprint. [Version 1] doi: 10.1101/2025.07.31.668042 (PMC12324431; doi:10.1101/2025.07.31.668042)
Supplement: Supplement 1 [file media-1.gz › supplemental_material/src/rscape_v2.6.0.tar.gz!/rscape_v2.6.0/lib/hmmer/documentation/userguide/inclusions/hit-serialize.pdf]

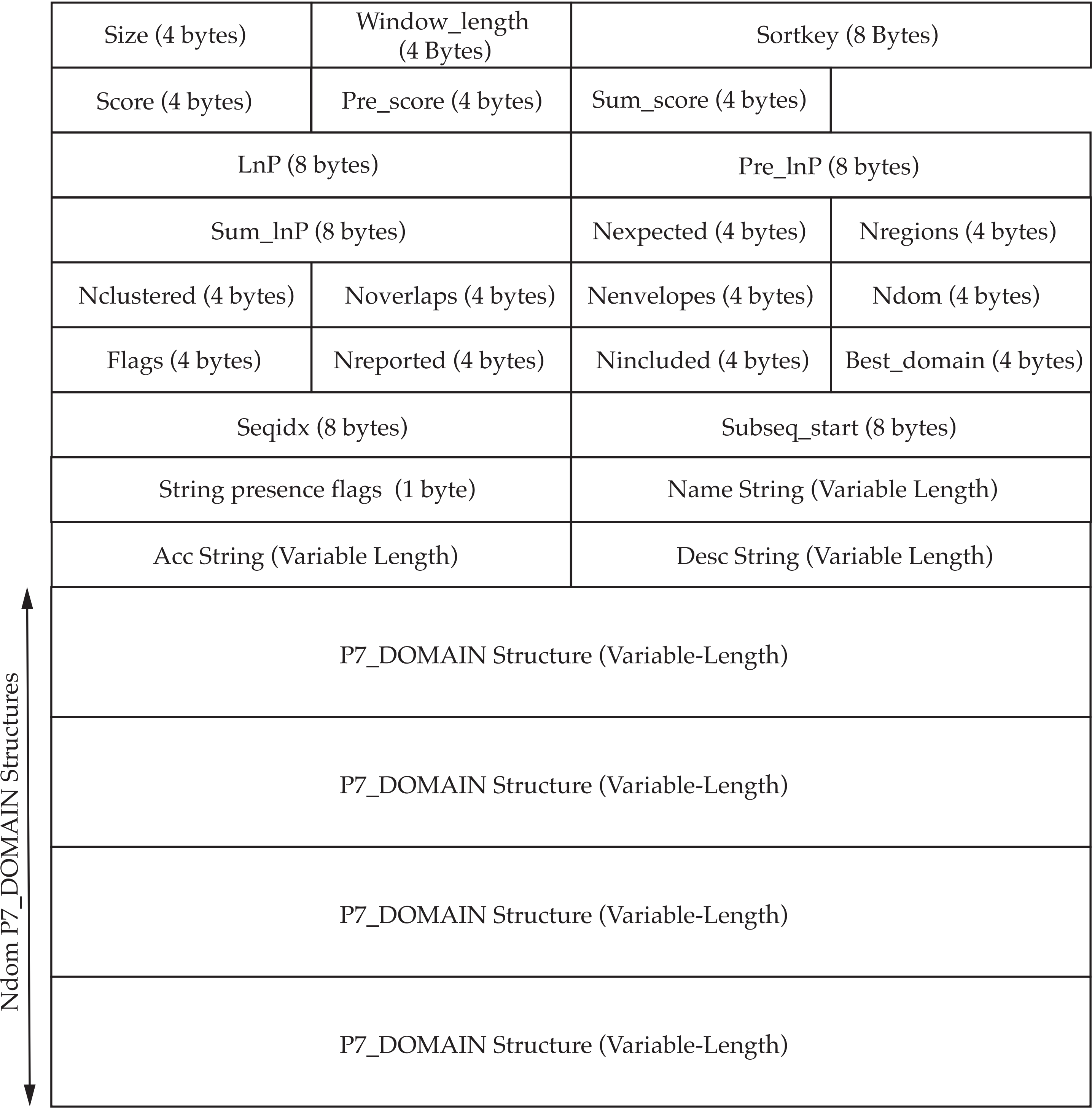
Size (4 bytes)
Window_length (4 Bytes)
Sortkey (8 Bytes)
Score (4 bytes)
Pre_score (4 bytes)
Sum_score (4 bytes)
LnP (8 bytes)
Pre_lnP (8 bytes)
Sum_lnP (8 bytes)
Nexpected (4 bytes)
Nregions (4 bytes)
Nclustered (4 bytes)
Noverlaps (4 bytes)
Nenvelopes (4 bytes)
Ndom (4 bytes)
Flags (4 bytes)
Nreported (4 bytes)
Nincluded (4 bytes)
Best_domain (4 bytes)
Seqidx (8 bytes)
Subseq_start (8 bytes)
String presence flags (1 byte)
Name String (Variable Length)
Acc String (Variable Length)
Desc String (Variable Length)
Ndom P7_DOMAIN Structures
P7_DOMAIN Structure (Variable-Length)
P7_DOMAIN Structure (Variable-Length)
P7_DOMAIN Structure (Variable-Length)
P7_DOMAIN Structure (Variable-Length)
